# Evaluating a Mobile Phone–Delivered Text Message Reminder Intervention to Reduce Infant Vaccination Dropout in Arua, Uganda: Protocol for a Randomized Controlled Trial

**DOI:** 10.2196/17262

**Published:** 2021-02-24

**Authors:** Daniel C Ehlman, Joseph Magoola, Patricia Tanifum, Aaron S Wallace, Prosper Behumbiize, Robert Mayanja, Henry Luzze, Joshua Yukich, Danni Daniels, Kevin Mugenyi, Fulgentius Baryarama, Nicholas Ayebazibwe, Laura Conklin

**Affiliations:** 1 Global Immunization Division Center for Global Health Centers for Disease Control and Prevention Atlanta, GA United States; 2 African Field Epidemiology Network Kampala Uganda; 3 Health Information Systems Program Uganda Kampala Uganda; 4 Uganda National Expanded Program on Immunization Ministry of Health Kampala Uganda; 5 Department of Tropical Medicine Tulane University School of Public Health and Tropical Medicine New Orleans, LA United States

**Keywords:** immunization, vaccination, reminder system, mHealth, short message service, text messages, cell phone, mobile phone, vaccination dropout, vaccination timeliness

## Abstract

**Background:**

Globally, suboptimal vaccine coverage is a public health concern. According to Uganda’s 2016 Demographic and Health Survey, only 49% of 12- to 23-month-old children received all recommended vaccinations by 12 months of age. Innovative ways are needed to increase coverage, reduce dropout, and increase awareness among caregivers to bring children for timely vaccination.

**Objective:**

This study evaluates a personalized, automated caregiver mobile phone–delivered text message reminder intervention to reduce the proportion of children who start but do not complete the vaccination series for children aged 12 months and younger in select health facilities in Arua district.

**Methods:**

A two-arm, multicenter, parallel group randomized controlled trial was conducted in four health facilities providing vaccination services in and around the town of Arua. Caregivers of children between 6 weeks and 6 months of age at the time of their first dose of pentavalent vaccine (Penta1; containing diphtheria, tetanus, pertussis, hepatitis B, and Haemophilus influenzae type b antigens) were recruited and interviewed. All participants received the standard of care, defined as the health worker providing child vaccination home-based records to caregivers as available and providing verbal instruction of when to return for the next visit. At the end of each day, caregivers and their children were randomized by computer either to receive or not receive personalized, automated text message reminders for their subsequent vaccination visits according to the national schedule. Text message reminders for Penta2 were sent 2 days before, on the day of, and 2 days after the scheduled vaccination visit. Reminders for Penta3 and the measles-containing vaccine were sent on the scheduled day of vaccination and 5 and 7 days after the scheduled day. Study personnel conducted postintervention follow-up interviews with participants at the health facilities during the children’s measles-containing vaccine visit. In addition, focus group discussions were conducted to assess caregiver acceptability of the intervention, economic data were collected to evaluate the incremental costs and cost-effectiveness of the intervention, and health facility record review forms were completed to capture service delivery process indicators.

**Results:**

Of the 3485 screened participants, 1961 were enrolled from a sample size of 1962. Enrollment concluded in August 2016. Follow-up interviews of study participants, including data extraction from the children’s vaccination cards, data extraction from the health facility immunization registers, completion of the health facility record review forms, and focus group discussions were completed by December 2017. The results are expected to be released in 2021.

**Conclusions:**

Prompting health-seeking behavior with reminders has been shown to improve health intervention uptake. Mobile phone ownership continues to grow in Uganda, so their use in vaccination interventions such as this study is logical and should be evaluated with scientifically rigorous study designs.

**Trial Registration:**

ClinicalTrials.gov NCT04177485; https://clinicaltrials.gov/ct2/show/NCT04177485

**International Registered Report Identifier (IRRID):**

DERR1-10.2196/17262

## Introduction

### Vaccination Coverage

Although global childhood routine vaccination coverage has increased markedly since the inception of the Expanded Program on Immunization (EPI) in 1974, coverage has plateaued since 2010, with rates of the third dose of diphtheria being between 84% and 86% [1]. According to Uganda’s 2016 Demographic and Health Survey (DHS), 49% of children aged 12 to 23 months received all recommended vaccinations by 12 months of age. Despite high coverage (95%) for the first dose of pentavalent vaccine (Penta1; containing diphtheria, tetanus, pertussis, hepatitis B, and *Haemophilus influenzae* type b antigens), which is given at 6 weeks of age, vaccination coverage for the third dose of pentavalent vaccine (Penta3), which is given at 14 weeks of age, was found to be 79% for a Penta1-Penta3 dropout of 17% [2]. Timely dosage of these vaccines remains low, threatening the health of Ugandan children with morbidity and mortality from vaccine-preventable diseases.

Innovative ways are needed to increase coverage, including increasing the recall rates of caregivers to bring children for timely vaccination [3]. Prompting health-seeking behavior via mobile technology interventions has been shown to improve health intervention uptake, particularly in high-income countries [4]. However, only a limited number of studies have evaluated the role of reminders sent by mobile phone text messages to increase vaccination coverage in low- and middle-income countries (LMICs).

### Background on Texting Reminders for Vaccination in LMICs

A few studies in LMICs [5-8] have assessed coverage improvements from the implementation of electronic immunization registers (EIRs) that had an automated text message reminder feature. The study designs varied in terms of scientific rigor and each concluded that coverage increased; however, it was not possible to assess the impact of text message reminders alone, as the EIRs provided the additional intervention of improving the tracking of individuals.

Other studies [9-14] enrolled participants between birth and Penta1 and followed them through Penta3, or in some cases through 12 months of age. Most of these studies focused on coverage at 2 to 4 weeks after the scheduled Penta3 date, as compared with the Kenya cluster randomized controlled trial (cRCT) [13], as it assessed coverage at 12 months of age, which is a typical EPI indicator. This cRCT study did not find the text message reminder intervention alone to significantly improve vaccination coverage at 12 months of age, likely due to high coverage in the control group. However, other studies concluded that text message reminders significantly improved vaccination coverage.

In addition, a few studies have focused primarily on the acceptability of text message reminders for vaccination [15-17]. Crawford et al [15] and Brown et al [16] found the acceptability to be high (99% and 95%, respectively); however, one study in Nigeria [17] found that only 69% of caregivers were willing to receive text message reminders.

Despite a body of research focused on vaccination reminders, there is still a deficit of scientifically rigorous studies evaluating the impact and scalability of text message reminders in LMICs. With 74% of households reported to have mobile phones [2], this research builds upon a growing mobile health (mHealth) system in Uganda to use personalized, automated text messages to remind caregivers of upcoming vaccination visits, which is hypothesized to reduce vaccination dropout (defined as starting but not completing the recommended vaccination schedule) in children under 12 months and to eventually contribute to reductions in morbidity and mortality due to vaccine-preventable diseases. With the goal of being scalable if shown to be effective, this intervention is designed to assess the impact of text message reminders using an mHealth platform already implemented in Uganda.

### Study Objectives

Primary objective:

- To evaluate a personalized, automated caregiver text message reminder intervention to reduce vaccination dropout for children aged 12 months and younger in select health facilities in Arua district

Secondary objectives:

- To measure the impact of a personalized, automated caregiver text message reminder intervention to increase the probability that children will receive Penta3 within 12 weeks of Penta1 receipt and MCV by 10 months of age.
- To assess caregiver acceptability of a personalized, automated caregiver text message reminder intervention
- To determine the cost-effectiveness of a personalized, automated caregiver text message reminder intervention from the provider (Ministry of Health [MOH]) perspective

## Methods

### Study Design

**Figure 1:**
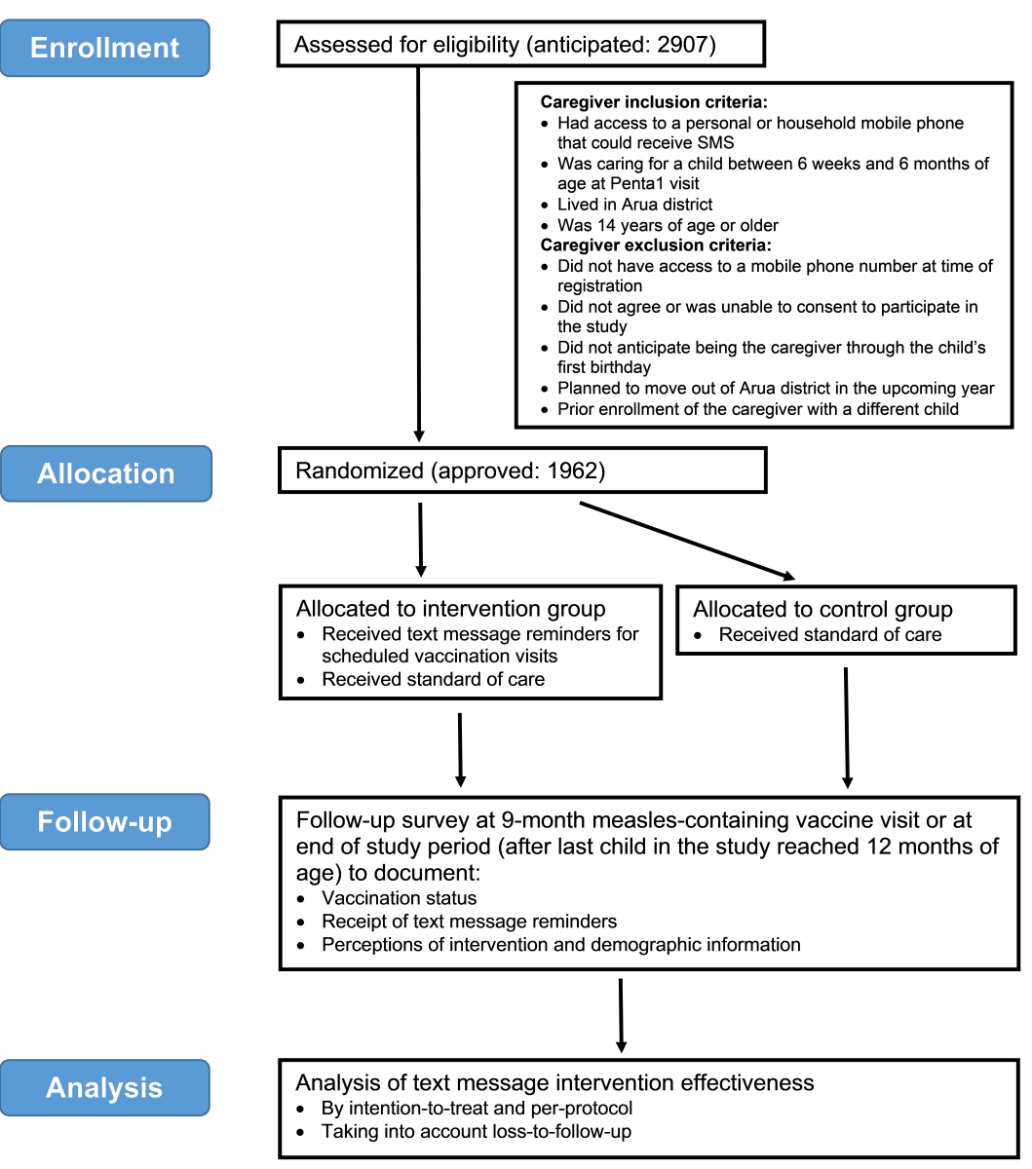
Diagram of study design.

A two-arm, multicenter, parallel group randomized controlled trial (RCT) was conducted in 4 health facilities providing vaccination services in and around the town of Arua. Caregivers were recruited at the time of their children’s Penta1 vaccination visit. Caregivers and their children were randomized to either the intervention arm or the control arm and followed until the exit interview, which took place at the health facility during the children’s measles-containing vaccine (MCV) visit or outside the health facility (generally at the caregiver’s home) after the last child in the study completed 1 year of age. If the original caregiver was not available at the exit interview, another caregiver was consented and interviewed if available. The protocol was designed taking into consideration criteria described by the Consolidated Standards of Reporting Trials (CONSORT) [18], and the diagram of the study design is shown in Figure 1.

### Setting and Participants

Districts located in Uganda but outside the city of Kampala were considered for inclusion in the study if they had the following characteristics:

- Penta1 administrative coverage ≥80% (calculated as the number of children vaccinated with Penta1 vaccine divided by the total number of eligible children)
- Penta1-MCV administrative dropout rate ≥10% (calculated as the number of children vaccinated with the Penta1 vaccine minus the number of children vaccinated with MCV, divided by the number of children vaccinated with Penta1)
- Interest from District Health Office
- Both urban and rural populations

Among the districts under consideration, Arua district was selected as the study area because it had a Penta1 administrative coverage of over 80% and a Penta1-MCV dropout rate greater than 10%. We used this parameter for dropout because 10% is considered the programmatic threshold for unacceptable dropout [19]. In December 2014, we conducted a site visit to the health facilities in and around Arua town, where we found that 18 out of 25 caregivers (72%) either had mobile phones with them or knew the phone numbers of their family phones. In addition, most caregivers indicated that they had village-level mobile phone reception as well as an electrical source to charge their phones (most typically solar charge).

Arua district is in northwest Uganda and borders the Democratic Republic of Congo in the west. Arua has an estimated population (2014) of 782,077, with an under 1-year-old target population of 28,605 [20]. Arua has 72 health facilities with varying levels of health services that are provided, including 3 hospitals.

To maximize study personnel efficiency, the 4 largest health facilities in and around Arua town were approached, and they agreed to participate as enrollment sites. Of these 4 facilities, 2 vaccinated every day, 1 vaccinated twice per week, and 1 vaccinated once per week. All 4 facilities provided outreach vaccination on an irregular basis.

During the enrollment period (February-July 2016), all caregivers who attended one of the selected health facilities for their children’s Penta1 vaccinations were approached to determine if they met the inclusion criteria and were willing to participate.

Caregiver participant inclusion criteria were as follows: had access to a personal or household mobile phone that could receive text messages; caregiver for a child between 6 weeks and 6 months of age at the time of Penta1 vaccination visit; lived in Arua district; and was 14 years of age or older.

Caregiver participant exclusion criteria were as follows: did not have access to a mobile phone number at the time of enrollment; did not agree or was unable to consent to participate in the study; did not anticipate being the caregiver through the child’s first birthday; planned to move out of Arua district in the upcoming year; prior enrollment of the caregiver with a different child.

Illiterate caregivers who were interested in participating, gave informed consent, and met the other inclusion criteria were enrolled. The understanding was that illiterate participants would have family members or friends read the text messages to them.

### Study Arms

The study consisted of 2 arms: a control arm and an intervention arm. The control arm received the standard of care in the selected health facilities, defined as health workers providing child vaccination home-based records (HBRs), known as Child Health Cards in Uganda, to caregivers, as available and providing verbal instruction of when to return for the next visit. The intervention arm received the standard of care as well as personalized, automated text message reminders sent to participants for each of their 3 subsequent vaccination visits, as per the Uganda National EPI schedule [21] (Table 1).

**Table 1:** Uganda National Expanded Program on Immunization schedule in 2016-2017.

Vaccination visit	Age	Vaccines
0	Birth	Bacillus Calmette-Guerin Vaccine OPV^a^ birth dose
1	6 weeks	Penta1^b,c^ OPV1 PCV1^d^
2	10 weeks	Penta2 OPV2 PCV2
3	14 weeks	Penta3 OPV3 PCV3 IPV^e,f^
4	9 months	MCV^g^

^a^OPV: oral polio vaccine.

^b^Penta1: pentavalent vaccine first dose.

^c^Pentavalent vaccine contains diphtheria, tetanus, pertussis, hepatitis B, and *Haemophilus influenzae* type b antigens.

^d^PCV1: pneumococcal conjugate vaccine first dose.

^e^IPV: inactivated polio vaccine.

^f^IPV was introduced in April 2016.

^g^MCV: measles-containing vaccine.

### Enrollment, Randomization, and Blinding

**Figure 2:**
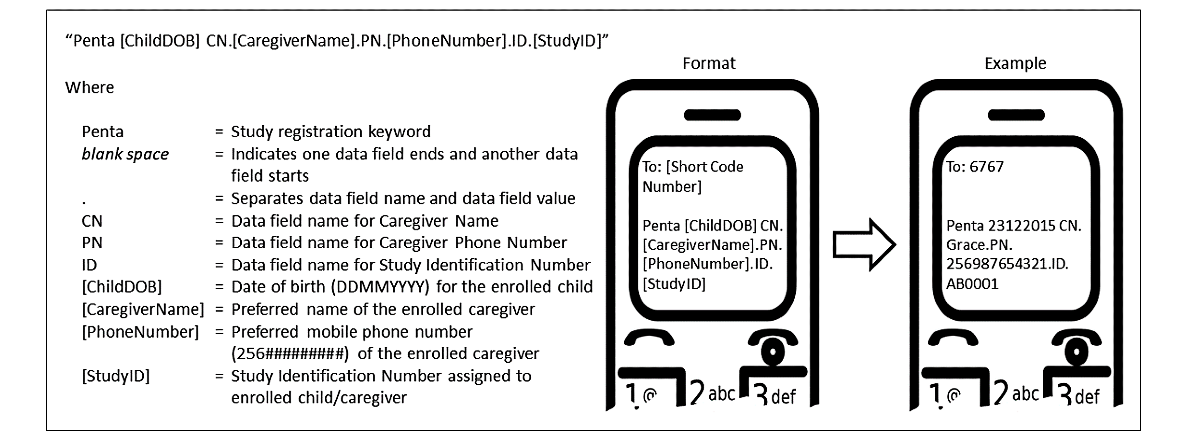
Screenshot of mobile phone displaying data registration format used at health facility study sites in Arua, Uganda.

Study personnel were stationed in the 4 selected health facilities and recruited caregivers of children between 6 weeks and 6 months of age who presented for their Penta1 vaccinations. Study personnel explained the study, obtained consent, and enrolled the eligible caregivers who agreed to participate in the study. Study participants responded to a brief preintervention questionnaire to collect basic demographic information and locating information. The health facility staff vaccinated the children, filled out the health facility immunization register, and filled out the children’s HBRs per standard practice in Uganda [22]. For each study participant, the study personnel sent a text message to a designated short code number with information that included the caregiver’s preferred first or last name, caregiver’s mobile phone number, child’s date of birth, and the participant study ID in the following format: “Penta [ChildDOB] CN.[CaregiverName].PN.[PhoneNumber].ID. [StudyID]” (Figure 2).

The information in the text message populated a centralized immunization registry database in a District Health Information System 2 (DHIS2) Software (version 2.26) Tracker Module instance created for this study. In addition, the mobile phone number of the study personnel who registered the study participant and the date that the registration text message was sent (which also served as the date of Penta1 vaccination) were recorded in the database. At the end of each day, study participants who were registered that day were randomized by computer using stratified permuted block randomization to either the control or the intervention arm, which was then recorded in the database as well. For randomization assignment, study participants were matched in order of registration to a sequence generated by nonfield study personnel before the start of enrollment. Stratification and blocking occurred at the health facility level; blocks of random size were used (lengths=2, 4, and 6) to minimize the ability to predict the next assignment. The health worker, study personnel, and participants were blinded to the randomization at assignment. However, unblinding of the intervention group occurred when they received the first text message reminder. The control group became unblinded, as they realized that they were not receiving text message reminders. On occasion, study participants shared their intervention status with health workers and field-level study personnel, thus unblinding them to the status of some caregivers. Nonfield staff study personnel who managed the database had access to intervention status after randomization assignment.

### Texting Reminder Intervention

Following the Uganda EPI schedule and accounting for health worker practices in Arua, the DHIS2 Tracker instance was programmed to automatically schedule follow-up vaccination visits at 30 days after Penta1 (for the visit to receive Penta2, oral polio vaccine [OPV] dose 2, and pneumococcal conjugate vaccine [PCV] dose 2), 61 days after Penta1 (for the visit to receive Penta3, OPV3, and PCV3), and 274 days after the child’s date of birth (for MCV). For each scheduled follow-up vaccination visit, the DHIS2 Tracker queued text message reminders to be sent on 3 different dates. At 7 AM on the scheduled dates, the DHIS2 Tracker delivered the messages to an SMS text message aggregator in Uganda, which then routed the messages to the appropriate Uganda telecommunications service providers, which in turn sent the messages to the appropriate participants. Every intervention group participant received the reminder in both English and Lugbara (the most common local language spoken in Arua). Penta2 text message reminders were sent 2 days before, on the day of, and 2 days after the scheduled vaccination visit. Penta3 and MCV text message reminders were sent on the scheduled day of vaccination and five and seven days after. Compared with the Penta2 text message reminders, the Penta3 and MCV text message reminders were sent later because at the time of reminder scheduling, the exact due dates for Penta3 were unknown (because Penta2 had not yet been received) and MCV (because DHIS2 Tracker could only schedule in terms of days, not months). Later reminders were preferred to reduce the possibility that caregivers would present too early for vaccination. As an automated intervention, text message reminders were sent regardless of whether the caregiver had already visited the health facility. Ideally, the visit for Penta3, OPV3, and PCV3 should have been scheduled 30 days after the visit for Penta2, OPV2, and PCV2. However, as the DHIS2 Tracker instance was not updated after vaccination visits, we had to schedule the visit in reference to the date of the visit for Penta1, OPV1, and PCV1.

Before study enrollment, consensus decision making with caregivers and health workers in Arua district and partners at the national level took place to finalize the pattern, timing, and exact wording of the text messages (Table 2). As some text message reminders would be automatically sent to caregivers either before the child was due for vaccination or after a completed visit, we emphasized in the text messages that the caregiver refer to the child’s HBR to confirm the date of the next vaccination.

**Table 2:** Content and timing of mobile phone text message reminders sent to intervention group participants’ mobile phones.

Vaccination visit, scheduled vaccination visit^a^, and message timing			Message^b^
**Vaccination visit 2: Penta^c,d^ dose 2; OPV^e^ dose 2; PCV^f^ dose 2**			
	**30 days after vaccination visit 1 (Penta1, OPV1, and PCV1)**		
		2 days before scheduled visit	“[CaregiverName], please bring your child for immunisation this week. Confirm the date in your child health card.”
		On the day of the scheduled visit	“[CaregiverName], don’t forget to immunise your child this week. Confirm the date in your child health card.”
		2 days after the scheduled visit	“[CaregiverName], don’t forget to immunise your child this week. Confirm the date in your child health card.”
**Vaccination visit 3: Penta3, OPV3, PCV3**			
	**61 days after vaccination visit 1 (Penta1, OPV1, and PCV1)**		
		On the day of the scheduled visit	“[CaregiverName], please bring your child for immunisation. Confirm the date in your child health card.”
		5 days after the scheduled visit	“[CaregiverName], please bring your child for immunisation. Confirm the date in your child health card.”
		7 days after the scheduled visit	“[CaregiverName], please bring your child for immunisation. Confirm the date in your child health card.”
**Vaccination visit 4: measles-containing vaccine**			
	**274 days after the child’s date of birth**		
		On the day of the scheduled visit	“[CaregiverName], please bring your child for measles immunisation this week. Confirm the date in your child health card.”
		5 days after the scheduled visit	“[CaregiverName], don’t forget to immunise your child against measles. Confirm the date in your child’s health card.”
		7 days after the scheduled visit	“[CaregiverName], don’t forget to immunise your child against measles. Confirm the date in your child’s health card”

^a^Vaccination visits subsequent to the Penta1 visit were scheduled in the District Health Information System 2 Tracker at the time of registration. As such, vaccination visit 3 (Penta3, OPV3, and PCV3) was scheduled based on the date of vaccination visit 1 (Penta1, OPV1, and PCV1).

^b^Messages were sent in both English and Lugbara.

^c^Penta: pentavalent vaccine.

^d^Penta contains diphtheria, tetanus, pertussis, hepatitis B, and *Haemophilus influenzae* type b antigens.

^e^OPV: oral polio vaccine.

^f^PCV: pneumococcal conjugate vaccine.

### Data Collection

Data were collected using a variety of tools: pre- and postintervention questionnaires, immunization data extraction form, and health facility record review form. At the time of enrollment, study personnel administered the preintervention questionnaire to participants (at the Penta1 visit) to gather basic demographic and locating information. At the time of the MCV visit, study personnel administered the postintervention questionnaire to participants to gather information on the acceptability of the text message reminder intervention, knowledge, attitudes, and beliefs regarding vaccination practices, and demographic information. Participants who did not return for MCV were interviewed at their homes. In some cases, the study personnel met the participants in another location that was convenient to them and reimbursed them for their transportation costs. In some other cases, the study personnel interviewed participants by phone because it was not feasible to interview in person. As part of the postintervention questionnaire, study personnel reviewed the HBRs of the child participants to extract vaccination data.

Every day during the enrollment period, study personnel completed the health facility immunization system record review form to capture service delivery process indicators (eg, vaccination sessions held, antigens administered). In addition, study personnel extracted vaccination data on the child participants from the immunization register.

### Data Management and Analysis

Initially, data collectors completed the participant interviews on paper and then entered the data into a REDCap electronic capture tool [23] on a tablet. After becoming comfortable with the tablet, data collectors entered the data directly into the tablet. Data were stored on a cloud-based server that used a 256-bit encryption. The server backed up the data daily and was only accessible through a secure password-protected website, which had individual log-ins only for authorized users. Data were removed from the server once the project was completed.

Data quality control was addressed at multiple stages. The first stage of quality control was the data collectors themselves. Adequate training minimized the risk of procedural errors. Furthermore, questionnaires were formatted electronically, which limited the amount of missing data, as logic patterns required the study personnel to enter all required fields. The electronic tools required the data collector to input values for each field. The second level of control was the local study coordinator. During data collection, the study coordinator ensured proper sampling and interviewing through daily periodic spot checks during data gathering. The study coordinator reviewed the data entered by the data collectors and then locked each record, after which it was no longer modifiable by the data collectors. The third level of control was the principal investigator who reviewed the data to ensure that (1) the sample size was reached, (2) the eligibility requirements of each participant were met, and (3) blanks or partially completed forms were minimized.

For the primary objective, a logistic regression model with fixed effects at the health facility level will be used to analyze differences between intervention and control groups in Penta3 and MCV coverage at 12 months of age. For the assessment of timeliness, a logistic regression model with fixed effects at the health facility level will be used to analyze differences between intervention and control groups in Penta3 coverage 12 weeks after receiving Penta1 and MCV coverage at 10 months of age. In addition, Cox proportional hazards models with fixed effects at the health facility level will be used to analyze time-to-event (ie, timeliness) outcome data. The primary analyses will be based on intention-to-treat, but per-protocol analysis will also be conducted. Dates of vaccination from HBRs will be used for analysis, supplemented with dates from the immunization register extraction if HBR dates are unavailable. In addition to logistic regression, log-binomial regression models will be used to estimate risk ratios. Data will be analyzed using statistical software such as STATA, SAS, or R.

### Sample Size

On the basis of administrative data from 2013 to 2014, we assumed a 16% Penta1-MCV dropout rate for the nonintervention group and calculated a sample size with a power of 90% and confidence level of 95% with the ability to detect a 5% decrease in dropout rate in the intervention group compared with the control group. A one-sided test was used for the sample size calculations.

Anticipated coverage of the control group was as follows: Penta1: 100% (Penta1 is an eligibility requirement); MCV: 84% (16% dropout).

Anticipated coverage of the intervention group was as follows: Penta1: 100% (Penta1 is an eligibility requirement); MCV: 89%.

We calculated the sample size based on the following formulas [24]:



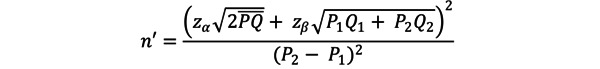



and



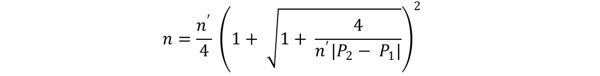



where *n’* is the sample size of each group if we ignore the continuity correction; *n* is the sample size of each group, accounting for the continuity correction; *P_1_* is the proportion found in the first sample (0.84); *P_2_* is the proportion found in the second sample (0.89); *Q_1_* is 1 minus *P_1_* (0.16); *Q_2_* is 1 minus *P_2_* (0.11); 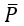 is the average of *P_1_* and *P_2_* (0.865); 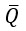 is the average of *Q_1_* and *Q_2_* (0.135); *z_α_ is* the z-score for a one-sided test with a level of significance of .05 (1.645); *z_β_ is* the z-score for power of 90% or *z_0.10_* (1.28).

To detect a change in MCV coverage, the sample size per arm (without attrition) needed was 838. Of these 838 per arm, we anticipated a 10% loss to follow-up for caregivers who do not return to the same health facility for MCV and who cannot be found at the end of the study or who have lost their HBRs. In addition, for the purposes of quality control, approximately 100 participants (about 5%) were recruited and contacted by mobile phone after their scheduled visits to assess the reliability of the intervention at sending text messages and having those messages received by the intended individual. Thus, an adjusted sample size of 1962 participants was necessary for enrollment.

Among caregivers approached during their children’s vaccination visit 1 (Penta1, OPV1, and PCV1), we anticipated an eligibility rate of 75% and a study refusal rate of 10%, thus estimating that study personnel would need to screen 2907 caregivers in order reach the sample size.

According to administrative data from the operational year 2013 to 2014, the 3 largest health facilities in and around Arua town administered a total of 9292 doses of Penta1 during the course of 1 year. On the basis of the number of caregivers that needed to be screened (2907) and the number of doses the 3 health facilities provided in a year (9292), we estimated that 16 weeks would be necessary for enrollment. After the first 2 months of study recruitment (February-March 2016), enrollment was found to be lower than expected, so a fourth health facility was added as a study site.

### Ancillary Research Objectives

In addition to the RCT study design to address the principal research objectives, we also conducted focus group discussions (FGDs) to more thoroughly assess caregiver acceptability of a personalized, automated caregiver text message reminder intervention. At the end of the study, study personnel conducted 8 FGDs with caregivers and spouses who were originally randomized into the intervention group from one of the select health facilities. A total of 4 types of FGDs were conducted:

1. Female caregivers who received messages on their personal phones and whose children were up to date with vaccinations
2. Female caregivers who received messages on their personal phones and whose children were not up to date with vaccinations
3. Female caregivers’ spouses who received messages on their personal phones and whose children were up to date with vaccinations
4. Female caregivers’ spouses who received messages on their personal phones and whose children were not up to date with vaccinations

The FGDs were audiorecorded, and 2 research assistants translated and transcribed (1 step) discussions into English. Thematic content analysis will serve as a strategic analytical approach. Codes will be identified and then refined through additional reviews of the data. The main themes will be identified, reviewed, further refined, categorized, and subcategorized; matrix analysis will be used to organize themes and assess patterns. Data will be analyzed using word processors, spreadsheets, and qualitative data analysis software.

In addition, we will conduct an economic analysis that will focus on evaluating the incremental costs and cost-effectiveness of the personalized, automated caregiver text message reminder intervention compared with standard practice. Costs will be evaluated from the provider (MOH) perspective and will include the start-up costs of developing the system and operational costs of delivering the text messages (including costs related to data charges for message deployment, system maintenance, and troubleshooting). For the calculation of incremental cost-effectiveness ratio (ICER), coverage data from the questionnaire will be an important determinant in evaluating the value for money in adopting the caregiver text message reminder strategy. Effectiveness will be measured by the cost per additional fully immunized child at 12 months of age in the intervention arm. Furthermore, we will estimate the costs of scaling up the proposed intervention by the MOH to cover the entire country using data on the expected catchment areas for health facilities in Uganda to determine the total cost of a nationwide system rollout. Sensitivity analysis will be conducted to determine how sensitive the ICER is to varying effectiveness and system costs.

### Ethical Considerations

The proposed research was minimal risk and did not present significant concerns regarding the ethical treatment of study participants. The protocol received institutional review board (IRB) approval by the Higher Degrees, Research and Ethics Committee at Makerere University, School of Public Health in Kampala, Uganda (HDREC 294), and was registered with the Uganda National Council of Science and Technology (SS 3924). The US Centers for Disease Control and Prevention relied on the Makerere University IRB (CDC Protocol #6721.0). The trial is registered with ClinicalTrials.gov (NCT04177485).

This research involved face-to-face interviews using questionnaires to determine vaccination status and assess knowledge, attitudes, beliefs, and perceptions of vaccination and text message reminders. There were no direct benefits for the participants. However, study results will be used to support evidence-based strategies to reduce vaccination dropout rates.

Written informed consent was obtained from the participants before data collection. Consent forms were available in both English and Lugbara. Study participants could include pregnant women (eg, an aunt caregiver with an eligible child may be pregnant with her own child) and emancipated minors (ie, mothers and fathers who are 14-17 years of age), but they were not the primary focus of this intervention. Study participants were provided details on how they could opt out of the study at any point and thus be removed from receiving text message reminders.

## Results

Pretesting of the preintervention questionnaire and the text message reminders occurred in November 2015. Pretesting of the postintervention questionnaire occurred in February 2016. Enrollment began in February 2016 and concluded in August 2016. Of the 3485 screened participants, 1961 were enrolled from a sample size of 1962. The preintervention questionnaire was administered during enrollment. Text message reminders were sent to the intervention group between February 2016 and April 2017. Follow-up interviews of study participants, including data extraction from the children’s vaccination cards and FGDs occurred between September 2016 and December 2017. A total of 8 FGDs were conducted and varied in size from 6 to 10 participants. Data extraction from the health facility immunization registers, completion of the health facility record review forms, and cost data collection occurred between January 2016 and September 2017. Data cleaning was completed. Logistic regression will test for differences between intervention and control groups; log-binomial regression and Cox proportional hazards regression will approximate the relative risk of vaccination and the hazard of timely vaccination, respectively, in the intervention group compared with the control group. The results are expected to be released in 2021.

## Discussion

Despite recent improvements in Penta1 in Uganda, coverage of both Penta3 and MCV coverage are suboptimal and indicate that 17% of children are not returning for immunization services later in infancy [2]. As vaccination coverage continues to get closer to 100%, it is increasingly difficult and expensive to continue to make improvements in coverage. Prompting health-seeking behavior with reminders has been shown to improve health service uptake in many contexts, including through the use of mobile technology. However, most of the evidence surrounding the effectiveness of these interventions is from high-income countries where mobile phone technology usage is generally more prevalent [4]. According to Uganda’s 2016 DHS, 46% of women and 66% of men aged 15 to 49 years owned a mobile phone in 2016 [2]. MOH-Uganda is interested in improving vaccination coverage, and Uganda has already been the site of many pilot projects that have attempted to use mobile technology to improve health. Faced with high rates of vaccination dropout, this research evaluates an intervention that uses text messages to remind mothers of upcoming vaccination visits, which is hypothesized to reduce vaccination dropout in children aged below 12 months and to eventually contribute to a reduction in morbidity and mortality due to vaccine-preventable diseases. The study has several possible limitations, including contamination of the control group participants through their interactions with intervention group participants, attrition bias due to incomplete data from loss to follow-up, and a study population that excluded households that did not have a mobile phone. In addition, although logistic regression is appropriate to test the hypotheses of interest, the resulting odds ratios are inadequate approximations of the relative risks.

As mobile phone ownership continues to grow in Uganda, their use in health interventions is both logical and practical. However, the implementation of mobile technologies in immunization service delivery needs to occur alongside methodologically rigorous evaluations that assess their effects on both immunization uptake and caregiver acceptability.

## Appendix

Multimedia Appendix 1 CONSORT-eHEALTH checklist (V 1.6.1).

## Abbreviations

cRCT: cluster randomized controlled trial
DHIS: District Health Information System
DHS: Demographic and Health Survey
EIR: electronic immunization register
EPI: Expanded Program on Immunization
FGD: focus group discussion
HBR: home-based record
ICER: incremental cost-effectiveness ratio
IRB: institutional review board
LMIC: low- and middle-income country
MCV: measles-containing vaccine
mHealth: mobile health
MOH: Ministry of Health
OPV: oral polio vaccine
PCV: pneumococcal conjugate vaccine
Penta: pentavalent vaccine
RCT: randomized controlled trial
